# An Algorithm of Association Rule Mining for Microbial Energy Prospection

**DOI:** 10.1038/srep46108

**Published:** 2017-04-10

**Authors:** Muhammad Shaheen, Muhammad Shahbaz

**Affiliations:** 1 Foundation University Rawalpindi Campus (FURC), Software Engineering, Islamabad, Pakistan; 2 University of Engineering and Technology (UET), Computer Science, Lahore, Pakistan

## Abstract

The presence of hydrocarbons beneath earth’s surface produces some microbiological anomalies in soils and sediments. The detection of such microbial populations involves pure bio chemical processes which are specialized, expensive and time consuming. This paper proposes a new algorithm of context based association rule mining on non spatial data. The algorithm is a modified form of already developed algorithm which was for spatial database only. The algorithm is applied to mine context based association rules on microbial database to extract interesting and useful associations of microbial attributes with existence of hydrocarbon reserve. The surface and soil manifestations caused by the presence of hydrocarbon oxidizing microbes are selected from existing literature and stored in a shared database. The algorithm is applied on the said database to generate direct and indirect associations among the stored microbial indicators. These associations are then correlated with the probability of hydrocarbon’s existence. The numerical evaluation shows better accuracy for non-spatial data as compared to conventional algorithms at generating reliable and robust rules.

There are certain microbes present in soil which are fed by hydrocarbons. The isolation of these microbes from soil may give some clues about the presence of hydrocarbons beneath earth’s surface. Such microbial anomalies have given some accurate results for hydrocarbon indication in past^1^. There exist a number of methods for microbiological prospecting of hydrocarbon. These methods include gas chromatography, carbon isotope analysis, counting methane, ethane and propane-oxidizing bacteria and number of colony forming units etc^2^,^3^. Most of the techniques used to determine effective microbes emphasize on cell count, number of colony forming units and activity level of hydrocarbon oxidizing bacteria in soil. A small number of statistical and data mining studies on this topic can be cited from literature. Blanch *et al*.^4^ used discriminant analysis, Carson *et al*.^5^ used both discriminant analysis and nearest neighbor algorithm for microbial source tracking. Brion *et al*.^6^ used artificial neural networks on watershed containing bacteria to measure the strength of gram-positive and negative bacteria. The bacteria are classified in gram positive and negative on the basis of physical properties of their cell walls and the presence of high level of peptidoglycan. Munoz *et al*.^7^ used statistical and inductive learning to develop predictive models for determination of microbial source. The growth in sizes of data repositories to terabytes increased number of experiments performed on these datasets for extracting interesting patterns. One of such data repositories is microbial data repository. The extracted patterns affect the decision about different things. Most of the microbial data sources are based upon the data collected by experimentation in chemical laboratories. Data mining relies on discovery of knowledge from huge repositories of data by analyzing data in historical perspective. As the amount of data increases, the reliability of patterns extracted from data increases^8^. It is a structured and modeled approach comprised of discrete and iterative steps, originating from raw data and concluding with patterns and predictions^8^,^9^. One of the popular data mining technique is association rule mining. Association rules are popular of its wider use on different types of data like numeric, ordinal, spatial and multimedia data etc. The basic form of association rule is that if an event X occurs it is more likely for event Y to occur. It is written as *X* → *Y* where X is the antecedent and Y is the consequent. Association rule *X* → *Y* is described with two measures, support and confidence. Support means that “how often the transactional records in database contain X and Y together” and confidence is a measure of accuracy of the rule^10^,^11^,^12^. This study is meant to provide modified form of an existing association rule mining algorithm i.e. context based positive and negative spatio temporal association rule mining (CBPNARM) to make it applicable on non spatial data. Secondly the algorithm is extended to be applied upon microbial data for microbial energy prospection. There does not exist an explicit list of microbial indicators on the basis of which microbial prospection may be made. This study selected list of indicators from existing literature and prepared a database for the same. The use of data mining techniques on microbial indicators for energy prospection is also not found in literature according to the best of our research. The use of association rule mining technique seems to be a promising approach to find correlation among microbial indicators and the effect of this correlation on prediction of new energy reserve. The mostly commonly used algorithm for association rule mining is Apriori algorithm^13^. This algorithm did not gaveany consideration to “context” which is an influencing variable. Context is the state of environment, entity and action^14^. Apriori algorithm extracts association rules among various microbial indicators without keeping in view the state of environment, entity and actions. It might be possible and seems to be usual that the microbial indications are caused by abnormal value of context variable hence misleading the explorist about energy prospection. What is required is to make those microbial indicators available at one platform which can contribute towards energy prospection. In the light of aforementioned this work contributes in the following two stages;

- An algorithm of association rule mining of spatial data based on context is recently developed in ref. 14. Since microbial data is not spatial in nature, for which an extension of the same algorithm for non-spatial data is proposed and applied on microbial data in this paper.
- Microbial indicators for energy prospection are explicitly identified and selected to prepare a relational database. Context based association rule mining is used for finding correlation among microbial indicators in the first phase and correlation of microbial indicators with hydrocarbon prospection in the lateral.

## Microbial Indicators

The oxidizing microbes found in the area above hydrocarbon reserves guides to build an energy prospecting method. Various surface indications may lead to the result-oriented analysis of such microbes^15^. Most of the reviews on microbial prospection of energy focused upon examining nutrients, physical state of oil, temperature, salinity and pressure^16^. One of the recent review on the topic was about the application of microorganisms in petroleum bioprocess and biosensors^17^. The microorganisms are good enough to be utilized for hydrocarbon prospection which have been discussed in refs 3,18, 19, 20. The diversity in bacteria is a broader topic covered in microbiology, however hydrocarbons are predominantly utilized by Brevibacterium, Corynebacterium, Flavobacterium, Mycobacterium, Nucardia, Pseudomonas and Rhodococcus^21^,^22^,^23^. In a study of 17 oil and gas fields, the success rate of microbial prospecting is 90% which means that 90% of the predictions based on microbial anomalies resulted in actual hydrocarbon wildcats. Despite the good results of study, the overall accuracy of microbial prospecting is not relied upon. Several microbial methods are found in literature which are mostly concerned with finding enumeration of cell contents in soil, measuring gas consumption rate, radioautography, microbial oil survey techniques (MOST), gas chromatography, carbon isotope analysis, counting methane/ethane/propane/butane oxidizing bacteria and strength of microbe colony forming units^2^,^3^,^24^. The surface manifestations and other microbial indicators are not explicitly listed at one place in the extant literature. The indicators given in Table 1 below is collected from refs 2,15,25,26 and stored along with data in a relational database. The determination of microbial anomaly and detailed description of these factors is beyond the scope of this work and widely available in the literature.

## Methods

### Context based association rule mining

The increase in horizontal and vertical sizes of data in repositories make databases an interesting laboratory for finding out hidden relationships among data attributes and data values^9^. In data mining, frequent associations in the dataset are found by using different algorithms of association rule mining but these rules sometimes mislead. Pure statistical measures are taken to find accuracy of a particular association rule. The accuracy of associations is even measured by statistical methods without making any spatial or non-spatial consideration. Hence the rules which are extracted in two different time intervals in the same vicinity are considered equally important and interesting without considering the underlying context in which the rules were extracted. For example, in microbial prospecting the maghemite can precipitate even because of salinity in water. So an association rule of the following form may not be valid.




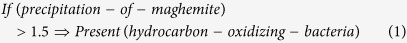




The rule seems to be valid and has been extracted by calculating Apriori frequent itmesets. But it might be possible that the precipitation of maghemite is increased because of water salinity. The associations are not considered if the corresponding item set is infrequent. The frequency of co-occurrence of an item set in a database might not be the only measure to find association rules. One or more than one context variables can change the scenario of resulting association rules. The same phenomenon is described in more detail in ref. 14. Different algorithms of association rule mining are proposed in literature. The algorithms which emerged context based positive and negative association rule mining (CBPNARM) algorithm are PNARM (positive and negative association rule mining)^10^ and Apriori algorithm^27^. To understand context based association rule mining for microbial databases, consider a microbial database MD, containing data of microbial indicators. The data is collected from different sites of potential energy reserves over multiple time periods. Let R = R1, R2, R3, …, Rn be the set of records contained in the database. Let T be the subset of items in R. Let T = T1, T2, T3, …, Tn are the time intervals on which the data is collected and site = site1, site2, site3, … siten are the sites from which the data is collected. Ti and Sitej represents ith and jth instances of time interval and site. Let context be the variable which stores the values of influencing parameter. The Apriori Algorithm of association rule mining^13^ is based on equations 1 and 2,^14^.

   For Positive Association Rules:
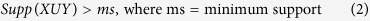


   For Negative Association Rules
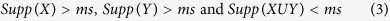


   Interestingness measure for both positive and negative association rule satisfies
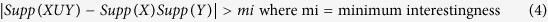


The total number of positive and negative association rules generated are pruned to remove uninteresting rules from rule set which may falsely be generated. Those rules which do not qualify interesting measures are removed, but there is no mechanism to deal with those rules which are falsely generated at some Ti because of an abnormal value of the context. The scenario is depicted in Table 2.

Association rules from the above table will be extracted on the basis of intersection of association rules derived at four different time intervals i.e. T1, T2, T3, T4. If the value of intersection >=3 then the rule is selected and pruned otherwise. In the scenario depicted in Table 2, association rules are extracted from a site at four different time intervals. The consequent of these rules predicts the presence of microbial prospect of energy at the given sites. The support value for these rules are given in the second column of the above table where the minimum support required is 3. In the last column of the table, value of context variable which is precipitation is given on each time interval. After extraction of all these rules at different time intervals, a final list of consolidated rules is prepared on the basis of intersection of the rule sets. The rules which are extracted thrice or more than this are included in final list. According to existing association rule mining algorithm, only Rule1 is included in the final list. Rule2 despite having good frequency is not included in the list because its support is 2 which is lesser than the value of minimum support (ms). Rule{2}/T{3} is different from Rule2/T1 and Rule2/T2, only in one term i.e. Pheh, <=X2. But it can also be seen in the table that the rule was derived under abnormal circumstances i.e. the value of context variable did not lie in its normal range which is 100–300. What it means is that because of abnormality in the value of context variable precipitation, the rule was falsely generated. Since the procedure of association rule mining was automatic for which the rule was not included in the final list. The example demonstrated the exclusion of a valid association rule from the list because of abnormal value of context variable. This establishes the need of consideration of context variable for generating valid association rules. The algorithm that we have used to mine positive and negative association rules from microbial data is an extension of CBPNARM which has been developed by^14^ in extension of refs 27,28. The existing algorithm with all its details is given in the author’s paper^14^. The extended form of the same is given in next section. Some of the definable terms used in the algorithm are given below^27^; A positive association rule 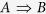 is of potential interest if

A and B are disjoint itemsets i.e., 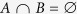

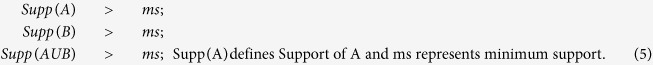

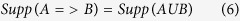

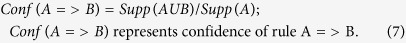
Similarly, the negative association rule *A* 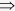 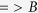 is of potential interest if

A and B are disjoint itemsets i.e. 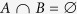



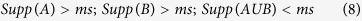

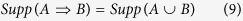

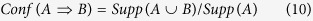


This will result in an exponential number of association rules which can be pruned by using the interestingness measure, as given in (eq. 7)^27^




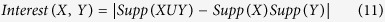







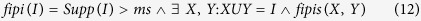




Where




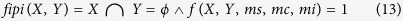




and




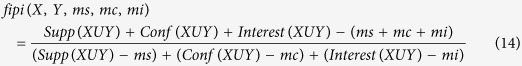




Fipi represents frequent itemset of potential interest. ms is the user specified minimum support, mc is the user specified minimum confidence, *ϕ* represents an empty set and mi is the user specified minimum interest. All these values of mi, ms and mc are selected by the domain expert based on the usual frequency of certain event. The interestingness measure is also incorporated in the support-confidence framework for negative association rule mining in the following way below^27^. J is an infrequent itemset of potential interest (iipis) if:




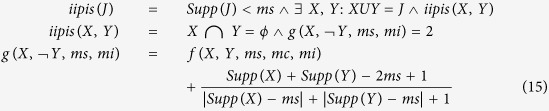




The selection of context variable is situation dependent. For example in the study of surface manifestations, rainfall, temperature, water level, vegetation viral attack etc can be considered as context variables. After the selection of context variables, we need to define normal range of the values for context variable. In our study the normal range is defined by two variables, an initial and final value to define context range named as CIV (context initial value) and CFV (context final value). Normal range means that any value below CIV or any value above CFVwere considered as “out of range” values. The selection of these two values is merely based upon one’s expertise in the field of study. In the example of Table 2 precipitation is considered as context variable whose normal range of values is between 100 and 300. In the highlighted entry of the table, 450 is an out of range value. In the method given in ref. 14, if the value of context variable lies in the normal range, both positive and negative association rules are mined as per normal procedure without making any change. If the value goes beyond either limit then there can be four possibilities detailed in Table 3. In our study, each of the possibility is handled by changing the value of support variable^14^.

In Table 3, Actual_support_value stores the value of support acquired by applying frequency introduced in Apriori algorithm^27^. Similarly, for positive association rules, “*difference*” stores the value of surplus added in support value. This surplus is added because of abnormal value of context. “*Difference*” in case of negative association rule stores the value of deficiency in support value because of abnormal value of context. The amended algorithm is given in the next section. The algorithm will mine positive and negative association rules^14^ from conventional microbial database in the light of context variable. The terminologies related to the algorithm are explained in finer details in ref. 14.

**Algorithm**




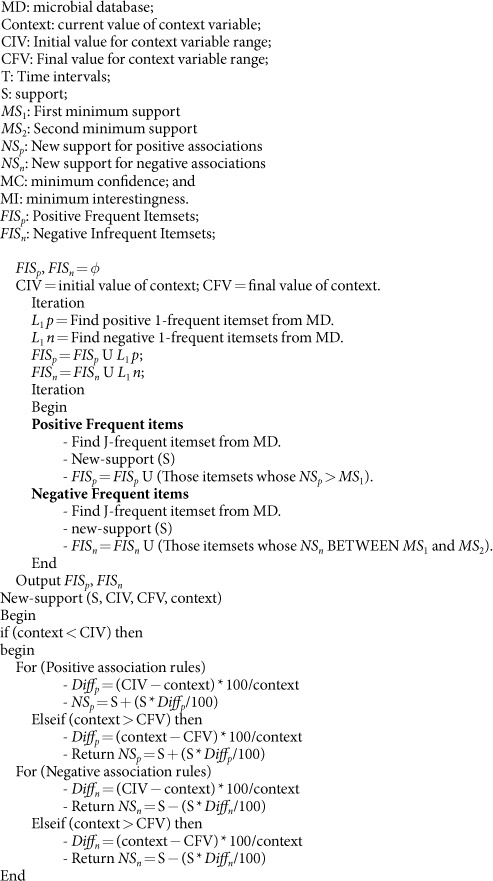




### Proposed Method

**Figure 1:**
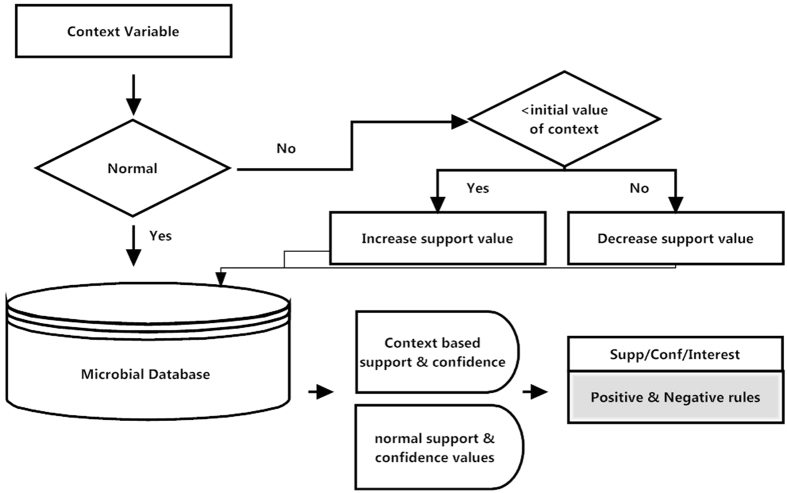
Process of context based association rule mining for microbial energy prospection.

**Figure 2:**
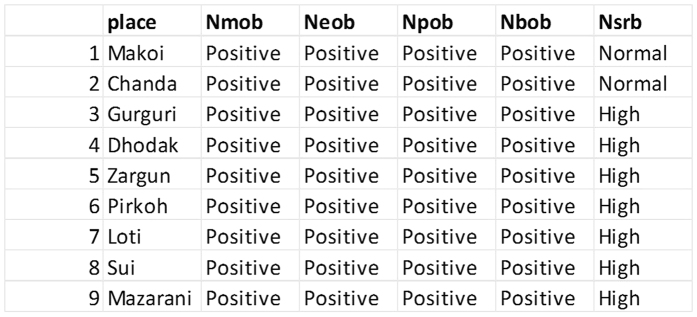
Data file for Microbial Prospection of Energy (before processing).

An algorithm for mining positive and negative association rules (PNARM) has already been devised by Wu *et al*.^27^ and implemented on spatial databases by Sharma *et al*.^28^. The extended form of the method after inclusion of context variable in association rule mining (CBPNARM) is proposed by ref. 14. The method added a variable i.e. “Context variable” to mine positive and negative association rules. This algorithm considers context variable to represent changing states of environment, time and situation based actions and different states of entities. Any change in the context variable changes the extracted association rule and this change, beyond a certain limit, invalidates the rule. The use of context variable in spatio temporal association rule mining is depicted in ref. 14. In this paper CBPNARM is further extended to include non-spatial association rules. The paper extended the existing work in two folds;

1. CBPNARM is extended to apply it on non-spatial databases.
2. An application of CBPNARM on microbial databases is presented as first application of the algorithm for microbial energy prospection. In this study, microbial data is collected from different sources and stored in a relational database. Apriori algorithm is applied to extract frequent item sets from the database. Three parameters of association rules are defined then, i.e. support, confidence and interestingness. The parameters are defined in section 3 and 4. On the basis of support and confidence, frequent item sets are extracted from the database. These rules also contain some rules which are not of potential interest. Such rules are eliminated on the basis of interestingness measure. Pruning of uninteresting association rules is done once in the above stated method. In the proposed technique, the rules are re oriented on the basis of context variable and pruned again on the basis of interestingness measure. For microbial data, salinity of water, temperature, humidity, rainfall and fossil pollution are considered as context variables. All the context variables are stored with CIV and CFV. The values of variables are re-adjusted according to the method given in Table 3. The readjustment of values would cause to generate a database with new values of microbial indicators and new set of association rules. The proposed method is given in detail in Figs 1 and 2. The microbial database of our study contains two tables. The structure of the tables is given in Table 4 and Table 5.

The values can be changed into ordinal values on the basis of the above mentioned ranges. The range values are stored in “Indicator_range_val” attribute of the database. The attribute “Prospect_coordinates” represents the latitude and longitude of the tested point in Degree, Minutes, and Seconds. The date and time on which the data is collected from the site is stored in “Date_of_evaluation”. As mentioned earlier, there are a total of five context variables stored in the database i.e. Temperature, Salinity of water, Humidity, Rainfall and fossil production. Table 5 contains only the list of indicators stored against unique Indicator_ID.

## Experiments and Results

The algorithm is implemented on data of multiple sites collected at different time intervals. The maps are geo-rectified then to convert images on to true lat/lon attributes. Spatial data is also converted to non-spatial form and stored in a conventional database in SQL Server. The reason for storage in conventional form is to ensure uniformity in all the datasets and testing of algorithm on non-spatial data. The data of 29 attributes given in Table 5 is stored in the database for 28 different sites of Pakistan. The data evolved over 178 different time intervals. 20 of these sites have energy. The size of database is small because of unavailability of all data. The values against all attributes are mapped in the database with an additional Boolean attribute indicating the result of prospection either in success or in failure. The same is illustrated in the Table 6. The algorithm proposed in Section 4 is executed then to find

1. Associations among microbial indicators and
2. Association between microbial indicator and result of exploration project in presence and absence of context variable.

**Figure 3:**
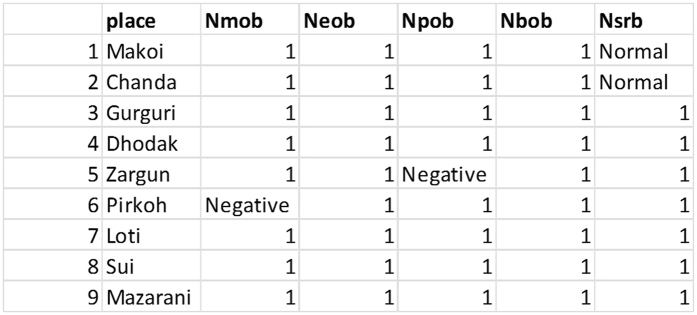
Data view of microbial indicators for prospect/non-prospect sites showing replacement of positive values with “1”.

**Figure 4:**
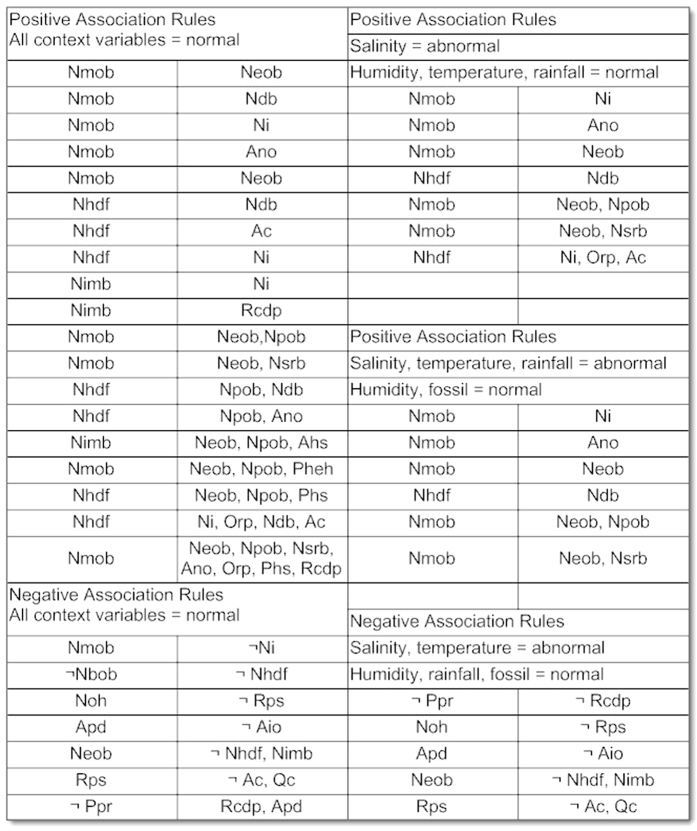
Positive and Negative Association Rule Mining in Microbial Datasets.

Once the data is mapped in the database, all positive values of attributes are replaced by 1 for ease of association rule mining. The attribute having value “1” is representing presence of energy reserve whereas every other value reflects absence. The same is depicted in Figs 3 and 4. Seven instances of the data for each of the context variable i.e. salinity of water, temperature, humidity, rainfall and fossil pollution are collected at the time when the value of these variables was not normal.

**Figure 5:**
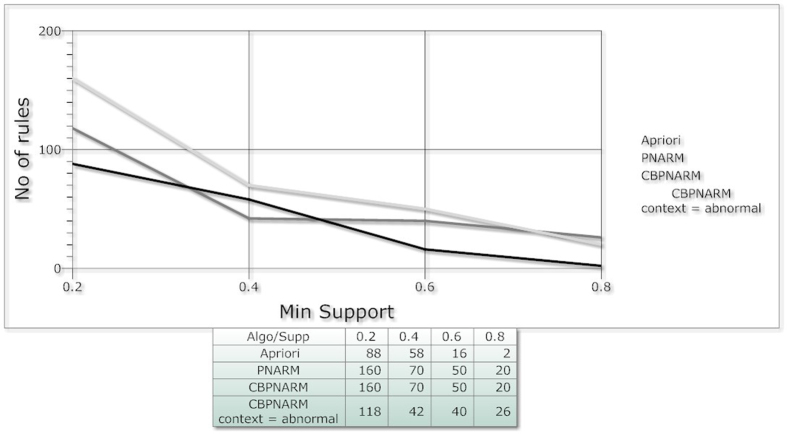
Plot; Number of rules with minimum support of Apriori, PNARM and CBPNARM.

The resulting association rules are organized in the database in such a form that each antecedent is mapped against all of its consequents up till 7-consequent. The association rules resulted from above scenario are shown in Fig. 5. Both the positive and negative association rules are mined by considering all the assumptions. The assumptions include presence of all context variables, absence of all context variables and absence of few. The positive rules contain positive antecedent and consequent while negative association rules may contain one or both of antecedents and consequents as negative. The figure indicates clear reduction in number of association rules when the context variable is considered abnormal. From the figure it can also be observed that an increase in number of context variables do not prune large number of association rules from existing rule set.

**Figure 6:**
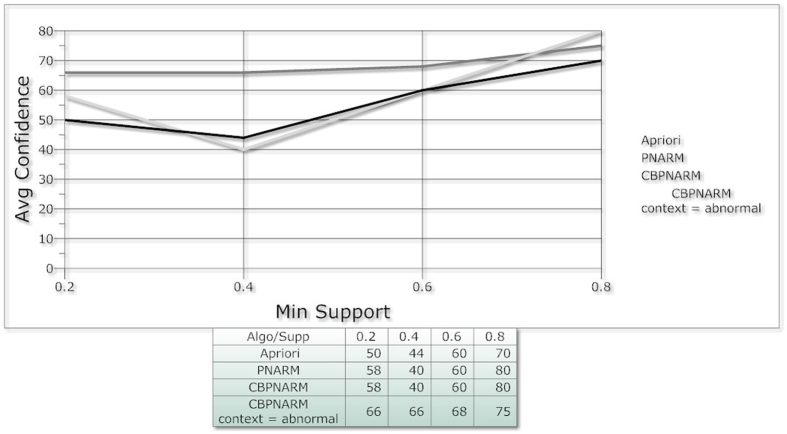
Min Support and avg confidence of Apriori, PNARM and CBPNARM.

**Figure 7:**
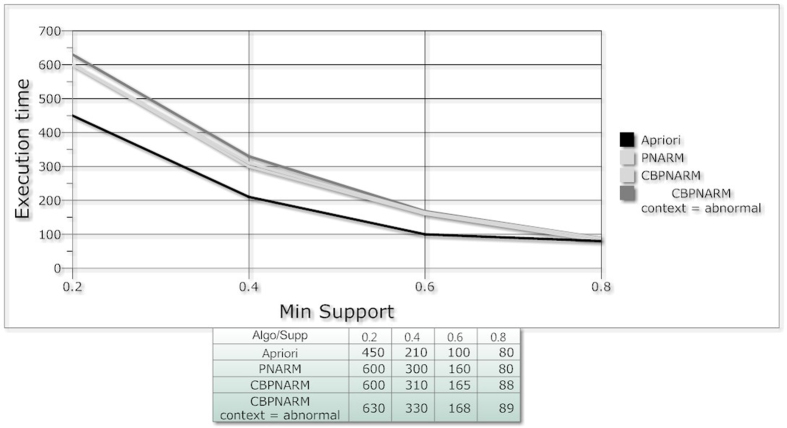
Plot; Min Support and Execution Time of Apriori, PNARM and CBPNARM.

As stated in ref. 14, once again we studied the results of our algorithm with respect to number of rules, average confidence of rules and total time taken to extract association rules (execution time). The comparison may not manifest the significance of the approach. The comparison is infact made to compare existing association rule mining approach (Apriori and PNARM) on non-spatial data to the proposed technique. These results are compared with the results produced by positive/negative Association rule mining algorithm (PNARM) and Apriori. In Fig. 5, the algorithms are compared on the basis of number of rules. PNARM always produces fewer rules than the proposed algorithm. Two cases of the proposed algorithm are considered in this plot (1). If the proposed algorithm is applied without taking in consideration in context variable (2). If the algorithm is executed by considering abnormal value of context variable. The algorithm produces equal number of rules with assumption (1) hence the line in the plot is not visible, whereas it produced comparatively fewer rules with the assumption (2) because, in that case, the value of the context variable exceeds the upper limit of the range. The insight brought by the plot of Fig. 5 can be evaluated against different aspects. The greater the number of association rules, the greater the patterns extracted from the database. Increase in support value causes the algorithm’s results to show a divergence towards a single point because there are very few co-occurrences in real datasets with much larger support. No of rules produced by apriori algorithm are lesser for most of the times because apriori algorithm do not consider negative rules. The results of PNARM lies above the line of CBPNARM with abnormal context value. The average number of rules in PNARM seems to be greater than number of rules extracted from CBPNARM but the significance of the CBPNARM algorithm can be viewed by analyzing both the plots of Figs 5 and 6 together. In the plot (Fig. 6), the confidence of rules extracted using our algorithm has the highest projection of all at various support values. The greater the value of confidence, the greater the accuracy of rules with the exception of the dependence of the variables. The confidence of rules gives a true projection up to a certain value of support because the increase in support returns very few co-occurrences from the data, hence limiting a broader capability to evaluate the algorithm. The confidence values of apriori rules is lesser than both PNARM and CBPNARM. CBPNARM produced rules with greater confidence up to 0.5 support when compared with the PNARM algorithm. The rules produced by PNARM are at the lowest confidence which demonstrates the significance of considering contextual information, especially for positive and negative spatial association rule mining—as far as PNARM produced the largest number of rules but with a smaller confidence value. The algorithm is an extended form of PNARM inheriting its total capability with an additional capability of context based mining. The execution time of the proposed algorithm is a bit higher as per our expectations. The additional module takes some extra time which seems to be negligible with the increase in minimum support. In Fig. 7, the three algorithms are compared on the basis of execution time. The evaluation of algorithm shows that it is more accurate in terms of granularity of output rules and confidence. Though the execution time of the algorithm is higher than the previous algorithms but the extracted patterns are decision-oriented, specific and clear. The increase in execution time is because of the inclusion of an external factor i.e. context which is not the part of the adopted procedural approach and can be considered as external influencing factor.

## Conclusion

An algorithm of context based positive and negative association rules mining is used on microbial datasets to predict potential energy sources. The algorithm showed improved accuracy in association rule mining with respect to the confidence, number of rules and execution time when compared with state of the art algorithms. Since the algorithm is generic and can further be generalized to be applied in different application domains. The domains of specific readers interest may be^29^,^30^,^31^,^32^,^33^,^34^ in which protein sequence analysis is done. The work can be extended further to automate context variable which yet needs users input. The automation of context variable will eliminate the need of expert’s input in selecting the type and value of context variable.

## Additional Information

**How to cite this article:** Shaheen, M. and Shahbaz, M. An Algorithm of Association Rule Mining for Microbial Energy Prospection. *Sci. Rep.* **7**, 46108; doi: 10.1038/srep46108 (2017).

**Publisher's note:** Springer Nature remains neutral with regard to jurisdictional claims in published maps and institutional affiliations.

## Figures and Tables

**Table 1:** Microbial Indicators^2^,^15^,^25^,^26^.

S. No	Name of Indicator	Abbreviation
1.	Number of methane oxidizing bacteria	Nmob
2.	Number of ethane oxidizing bacteria	Neob
3.	Number of propane oxidizing bacteria	Npob
4.	Number of butane oxidizing bacteria	Nbob
5.	Number of sulphate reducing bacteria	Nsrb
6.	Number of denitrifying bacteria	Ndb
7.	Number of interisitial	Ni
8.	Number of hydrocarbons dissolved in fluids	Nhdf
9.	Number of occluded hydrocarbons	Noh
10.	Number of adsorbed hydrocarbons	Nah
11.	Rate of carbon dioxide production	Rcdp
12.	Number of identified microscopic objects	Nimb
13.	Amount of hydrogen sulphide	Ahs
14.	Amount of nitrogen	An
15.	Amount of nitrogen oxide	Ano
16.	Amount of paraffin dirt	Apd
17.	Oxidation reduction potential (Eh)	Orp
18.	PH of the system	Phs
19.	PH/Eh ratio	Pheh
20.	Rate of precipitation of silica	Rps
21.	Amount of iron oxides	Aio
22.	Amount of phosphates	Ap
23.	Amount of carbonates	Ac
24.	Quantity of clays	Qc
25.	Precipitation of pyrite	Ppr
26.	Precipitation of greigite	Pg
27.	Precipitation of pyrrhotite	Ppy
28.	Precipitation of maghemite	Pmg
29.	Quantity of magnetic particles	Qmp

**Table 2:** Importance of context variable in association rules; Prec = Precipitation, Range = 100–300, S = support of rules, ms = minimum support.

Rules	Parameters	Context
Rule1/T1:is_a (Apd, >=X1)ˆis_a (Pheh, >=X2)ˆdiff_of (Aio.Rps, >X3)ˆcolor_of (soil, Y1) => found (MP, true)	S = 8, ms = 3	Prec = 250
Rule2/T1:is_a (Apd, >=X1)ˆis_a (Pheh, >=X2)ˆdiff_of (Aio.Rps, >X3)ˆcolor_of (soil, Y1)ˆis_a (Ni, >=X3) => found (MP, true)	S = 6, ms = 3	Prec = 250
Rule3/T1:is_a (Apd, >=X1)ˆis_a (Pheh, <=X2)ˆdiff_of (Aio.Rps, >X3)ˆcolor_of (soil, Y1) => found (MP, true)	S = 6, ms = 3	Prec = 450
Rule4/T1:is_a (Apd, >=X1)ˆis_a (Pheh, <=X2)ˆis_a (Aio, >X4)ˆcolor_of (soil, Y1) => found (MP, true)	S = 8, ms = 3	Prec = 370
Rule1/T2:is_a (Apd, >=X1)ˆis_a (Pheh, >=X2)ˆdiff_of (Aio.Rps, >X3)ˆcolor_of (soil, Y1) => found (MP, true)	S = 7, ms = 3	Prec = 250
Rule2/T2:is_a (Apd, >=X1)ˆis_a (Pheh, >=X2)ˆdiff_of (Aio.Rps, >X3)ˆcolor_of (soil, Y1)ˆis_a (Ni, >=X3) => found (MP, true)	S = 4, ms = 3	Prec = 250
Rule3/T2:is_a (Apd, >=X1)ˆis_a (Pheh, >=X2)ˆdiff_of (Aio.Rps, >X3)ˆcolor_of (soil, Y1) => found (MP, true)	S = 4, ms = 3	Prec = 250
Rule1/T3:is_a (Apd, >=X1)ˆis_a (An, >=X5)ˆclose_to (Prospect, rock) => found (MP, true)	S = 6, ms = 3	Prec = 250
Rule2/T3:is_a (Apd, >=X1)ˆis_a (Pheh, <=X2)ˆdiff_of (Aio.Rps, >X3)ˆcolor_of (soil, Y1)ˆis_a (Ni, >=X3) => found (MP, true)	S = 5, ms = 3	Prec = 450
Rule1/T4: is_a (Apd, >=X1)ˆis_a (Pheh, >=X2)ˆdiff_of (Aio.Rps, >X3)ˆcolor_of (soil, Y1) => found (MP, true)	S = 5, ms = 3	Prec = 250
Final Rule Set Rule1:is_a (Apd, >=X1)ˆis_a (Pheh, >=X2)ˆdiff_of (Aio.Rps, >X3)ˆcolor_of (soil, Y1) => found (MP, true)		

**Table 3:** Four Cases of Context Variable^14^.

	Cases (Context)	Proposed Change in Support
For Positive Rules	Context < CIV	Difference = (CIV − context) * 100/context; New_support_value = actual_support + (actual_support * Difference)/100
For Positive Rules	Context > CFV	Difference = (context − CFV) * 100/context; New_support_value = actual_support − (actual support * Difference)/100
For Negative Rules	Context < CIV	Difference = (CIV − context) * 100/context; New_support_value = actual_support − (actual support * Difference)/100
For Negative Rules	Context > CFV	Difference = (context − CFV) * 100/context; New_support_value = actual_support + (actual support * Difference)/100

**Table 4:** Microbial Prospection Database Structure.

No	Name of attribute	Data type	Length
1.	ProspectID	Numeric	10
2.	IndicatorID	Numeric	20
3.	Indicator Range	Numeric	10
4.	Prospect coordinates	DMS	8
5.	Date of evaluation	Date	8
6.	Context temp	Boolean	2
7.	Context salinity	Boolean	2
8.	Context humidity	Boolean	2
9.	Context rainfall	Boolean	2
10.	Context fossil	Boolean	2

**Table 5:** Microbial Indicator Table Structure.

S. No	Name of attribute	Data type	Length	Constraint
1.	IndicatorID	Numeric	20	Unique
2.	Indicator Name	String	30	NIL

**Table 6:** Summary of Benchmark.

1.	Total number of attributes in database	29
2.	Total number of records	4984
3.	Total number of sites	28
4.	Sites with positive prospection results	20
5.	Sites with negative prospection results	8
6.	Available spatial records (converted to non spatial)	5,81,504
7.	Available non spatial records	28
